# Noninvasive Imaging of the Immune Checkpoint LAG-3 Using Nanobodies, from Development to Pre-Clinical Use

**DOI:** 10.3390/biom9100548

**Published:** 2019-09-29

**Authors:** Quentin Lecocq, Katty Zeven, Yannick De Vlaeminck, Sandrina Martens, Sam Massa, Cleo Goyvaerts, Geert Raes, Marleen Keyaerts, Karine Breckpot, Nick Devoogdt

**Affiliations:** 1 Laboratory for Molecular and Cellular Therapy (LMCT), Vrije Universiteit Brussel, Laarbeeklaan 103, B-1090 Brussels, Belgium; quentin.lecocq@vub.be (Q.L.); katty.zeven@vub.be (K.Z.); yannick.de.vlaeminck@vub.ac.be (Y.D.V.); cleo.goyvaerts@vub.ac.be (C.G.); 2 Laboratory of Medical and Molecular Oncology (LMMO), Vrije Universiteit Brussel, Laarbeeklaan 103, B-1090 Brussels, Belgium; sandrina.martens@vub.be; 3 Unit of Cellular and Molecular Immunology (CMIM), Vrije Universiteit Brussel, Pleinlaan 2, B-1050 Brussels, Belgium; sam.massa@vub.ac.be (S.M.); geert.raes@vub.be (G.R.); 4 Myeloid Cell Immunology Lab, VIB Center for Inflammation Research Center, Brussels, Pleinlaan 2, B-1050 Brussels, Belgium; 5 In Vivo Cellular and Molecular Imaging Laboratory (ICMI), Vrije Universiteit Brussel, Laarbeeklaan 103, B-1090 Brussels, Belgium; Marleen.Keyaerts@vub.be; 6 Nuclear Medicine Department, UZ Brussel, Laarbeeklaan 101, B-1090 Brussels, Belgium

**Keywords:** nanobody, single domain antibody, immune checkpoint, LAG-3, molecular imaging, nuclear imaging, cancer, immune cell

## Abstract

Immune checkpoint inhibition (ICI) is a promising cancer therapy, which has progressed rapidly from a preclinical concept to clinical implementation. Commonly considered targets in ICI are CTLA-4, PD-1/PD-L1, and LAG-3, and the list grows. As ICI is generally only beneficial for a subset of patients, there is a need to select patients that are eligible for therapy as well as to monitor therapy response. There is growing interest to do this noninvasively, by molecular imaging with target-specific tracers. To this day, noninvasive imaging has focused on CTLA-4 and PD-1/PD-L1, while there is no noninvasive tool available to accurately assess LAG-3 expression in vivo. In this proof-of-concept study, we developed nanobodies, the smallest functional fragments from camelid heavy chain-only antibodies, to noninvasively evaluate mouse LAG-3 expression using single photon emission computed tomography (SPECT)/CT imaging. The in vitro characterization of 114 nanobodies led to the selection of nine nanobodies binding to mouse LAG-3. The injection of ^99m^Technetium-labeled nanobodies in healthy mice showed specific uptake in immune peripheral organs like the spleen and lymph nodes, which was not observed in LAG-3 gene knock-out mice. Moreover, nanobody uptake could be visualized using SPECT/CT and correlated to the presence of LAG-3 as assessed in flow cytometry and immunohistochemistry. SPECT/CT scans of tumor bearing mice further confirmed the diagnostic potential of the nanobodies. These findings substantiate the approach to use nanobodies as a tool to image inhibitory immune checkpoints in the tumor environment.

## 1. Introduction

Inhibitory immune checkpoints play an essential role in the control and limitation of T-cell activation and functionality. When functioning properly, these control mechanisms can prevent autoimmunity, excessive inflammation, and tissue damage. Tumor cells exploit inhibitory immune checkpoints to develop defense mechanisms against the host’s immune system. Together with the infiltration of regulatory T cells (Tregs), myeloid-derived suppressor cells (MDSCs), and type 2-activated tumor-associated macrophages (TAMs), the presence of inhibitory immune checkpoints creates a strong resistance that limits the effective elimination of tumor cells by the induction of a functional unresponsiveness [1].

Blockade of inhibitory immune checkpoints, such as CTLA-4 and PD-1/PD-L1, has changed the classical approach to treat cancer [2]. Since the FDA approval of therapeutics targeting CTLA-4 and PD1/PD-L1 substantial interest raised for ‘next generation’ immune checkpoints such as LAG-3 (CD223) [3]. As currently reported in the literature, LAG-3 expression has been detected on the surface of activated T cells, Tregs, B cells, natural killer (NK) cells, and plasmacytoid dendritic cells (DCs) [4]. LAG-3 is known to downregulate T-cell responses via interaction with major histocompatibility complex class-II (MHC-II), present on tumor cells or antigen-presenting cells like DCs [5]. Additionally, three novel LAG-3 ligands have been reported: galectin-3, liver sinusoidal endothelial cell lectin (LSECtin), and fibrinogen-like protein 1 (FGL1) which are abundantly present in the tumor environment to help regulate and effectively abolish anti-tumor immunity of CD8^+^ T cells [6,7,8]. LAG-3 expression is often observed on tumor-infiltrating lymphocytes (TILs) and is associated with shorter progression-free survival in patients treated with anti-PD-1 therapy, suggesting independence of these immune evasion pathways [9,10]. Preclinical research demonstrated that monoclonal antibody (mAb) mediated LAG-3 blockade activates antigen-specific T cells at the tumor site, ultimately leading to a disruption in tumor growth [11,12,13]. The latter instigated the development of LAG-3 blocking moieties suited for the human setting and recently resulted in the execution of phase I/II clinical trials by Bristol Myers-Squibb (BMS), Novartis, Merck and Macrogenics, making LAG-3 the third inhibitory immune checkpoint targeted with an antagonistic mAb in the clinic [14,15].

Unfortunately, many patients fail to respond to immune checkpoint inhibition (ICI). For example, an objective response to single agent blockade with the anti-PD-1 mAb nivolumab is observed in 21% of patients with renal cell carcinoma [16] and 45% with advanced melanoma [17]. Correlative studies that performed immunohistochemistry (IHC) on tissue biopsies suggest that PD-L1 expression observed on tumor-infiltrating immune cells or on cancer cells could serve as a predictive marker for PD-1 blockade treatment, resulting in the development of complementary or companion diagnostics for some treatment indications [18]. Nevertheless, detection of PD-L1 expression in IHC of tumor biopsies is no guarantee for therapy response since some patients with clear positivity can fail therapy while patients with no apparent PD-L1 expression can benefit from PD-L1 blocking treatment [19]. The latter can be explained by the high heterogeneity of inhibitory immune checkpoint expression in primary tumors and metastases that are furthermore likely to change over time. Additionally, IHC relies on biopsies, which are tempospatially-restricted samples of ever evolving heterogeneous tumor masses. Consequently, IHC is a less suitable technique to stratify patients or predict therapy outcome. Preclinical studies evaluating noninvasive imaging using positron-emission tomography (PET) or single-photon emission computed tomography (SPECT) with radiolabeled antibodies to CTLA-4, PD-1, or PD-L1, suggest that this imaging method could be a valuable approach to improve patient selection [20,21]. Early-phase clinical trial results corroborate these preclinical findings, showing that pretreatment PET signals are more reliable to predict therapy response than IHC-based evaluation [22,23].

The use of nanobodies (Nbs), the recombinant variable domains (VHH) of heavy-chain only antibodies (HCAbs) that are naturally present in animals from the *Camelidae* family, is increasingly explored in the field of immuno-oncology [24]. Their small size and unique epitope targeting possibilities make them very interesting to target proteins present in dense tissues like the tumor environment [24]. Additionally, they can be easily engineered and produced in prokaryotic systems or yeast cells [25,26]. Nbs have been studied for molecular imaging of inhibitory immune checkpoints like PD-1/PD-L1 and CTLA-4 in the tumor environment [27,28,29,30,31]. Overall, preclinical molecular imaging studies show that Nbs are excellent tools to visualize the heterogenic expression of inhibitory immune checkpoints. Moreover, Xing Y. et al. conducted a phase I clinical study evaluating the use of ^99m^Technetium (^99m^Tc) labeled Nbs for SPECT imaging of PD-L1 in patients with non-small cell lung cancer, showing that it is safe and feasible to image PD-L1 levels in the tumor as soon as 2 h after injection [29].

These results encourage the development of Nbs for molecular imaging of the inhibitory immune checkpoint receptor LAG-3. As this requires substantial preclinical evaluation, we developed and validated Nbs targeting mouse LAG-3 (moLAG-3) as probes for SPECT imaging. After alpaca immunization and biopanning via phage display, 114 Nbs were identified to bind recombinant LAG-3 protein. Bacterial extracts of these Nbs were further analyzed for binding to moLAG-3 using ELISA, flow cytometry and off-rate analysis using surface plasmon resonance (SPR). As such, nine moLAG-3 binding Nbs were selected. These Nbs were bacterially produced and purified, tested in flow cytometry, and their affinity was analyzed using SPR. Next, we labeled them with ^99m^Tc, after which the biodistribution was assessed in immunocompetent and moLAG-3 gene-deficient mice by SPECT/CT imaging and dissection analyses. Subsequently, we evaluated the specific targeting of these Nbs in mice harboring tumors modified to overexpress mouse LAG-3.

## 2. Materials and Methods

### 2.1. Reagents

Specific binding of hemagglutinin (HA) tagged Nbs from small-scale productions was detected by ELISA and flow cytometry using an anti-HA antibody (Biolegend, clone 16B12). For flow cytometry a phycoerythrin (PE) labeled anti-mouse IgG antibody (BD biosciences, clone A85-1) was used to visualize Nb binding. For ELISA, the primary anti-HA antibody (Biolegend, clone 16B12) and the secondary anti-mouse IgG1κ (alkaline phosphatase conjugated) was used to detect mouse LAG-3 (moLAG-3) binding Nbs. An anti-His antibody (AbD Serotec, clone AD1.1.10), was used in flow cytometry to detect binding of purified hexahistidine (His_6_) tagged Nbs from large-scale productions. A PerCP-eFluor 710 or PE labeled antibody specific for moLAG-3 (Biolegend, clone eBioC9B7W) was used in flow cytometry to evaluate moLAG-3 expression on cells. The antibodies used to discriminate immune populations in flow cytometry were CD45.2-APC-eF780 (BD Biosciences, clone 104), CD11b-AF700 (BD Biosciences, clone M1/70), Ly6G-AF647 (BD Biosciences, clone 1AB), MHCII-PE-Dazzle 594 (Biolegend, clone M5/114.15.2), CD11c-AF488 (BD Biosciences, clone HL3), F4/80-BV421 (BD Biosciences, clone 6F12), Ly6C-PE-Cy7 (BD Biosciences, clone AL-21), CD19-FITC or AF647 (BD Biosciences, clone 1D3), CD335-AF647 (Biolegend, clone 29A1.4), CD160-PE-CF59 (BD Biosciences, clone CNX46-3 4), CD3-PE-Cy7 (Biolegend, clone 17A2), CD4-AF700 (BD Biosciences, clone RM4-5), CD8a-BV450 (BD Biosciences, clone 53-67), CD25-BB515 (BD Biosciences, clone PC61). Recombinant mouse and human LAG-3-Fc fusion proteins (RnDsystems, 3328-L3 and 2319-L3) were used for immunization, biopanning, ELISA, and SPR. T-activator CD3/CD28 dynabeads (11456D, Thermo Fisher Scientific) were used to stimulate spleen suspensions.

### 2.2. Isolation of LAG-3-Specific Nbs

Two llamas were subcutaneously immunized six times weekly, each time with a mixture containing 100 μg recombinant mouse and human LAG-3-Fc proteins (RnDsystems, cat. 3328-L3 and 2319-L3). Gerbu LQ#3000 was used as adjuvant. On the day 40, blood was collected for lymphocyte preparation from which total RNA was isolated. Using oligo(dT) primers, cDNA was synthesized from VHH encoding sequences using PCR. These amplicons were used as a source to create two Nb-phage display libraries in the pMECS phagemids as described previously [32]. These libraries were phage-displayed and subjected to four rounds of biopanning on moLAG-3 recombinant protein that was immobilized on immunosorbent plastic. Small-scale freeze-thaw periplasmic extracts of individual clones were made and tested in ELISA on moLAG-3 and control-Fc fusion proteins. Different moLAG-3-specific Nb clones were identified by sequencing.

### 2.3. Bacterial Production, Purification, and Quality Control of Nbs

Nb production and purification is detailed elsewhere [32]. Briefly, the sequences of selected Nbs, were PCR amplified using PCR with primers A6E (5′ GAT GTG CAG CTG CAG GAG TCT GGG GGA GG 3′) and PMCF (5′ CTA GTG CGG CCG CTG AGG AGA CGG TGA CCT GGG T 3′). cDNA of selected Nbs was cloned into PstI/BstEII digested pHEN6 plasmid to encode a C-terminal HIS_6_ tag. Selected Nb clones in pHEN6 were transformed into WK6 *Escherichia coli* (*E. coli*) for large-scale production. Each clone was cultured in 1 L supplemented terrific broth (TB) medium. Periplasmic extracts were generated by osmotic shock with Tris EDTA sucrose (TES) solution. The extracts were incubated with 1 mL of HisPur™ Ni-NTA Resin (ThermoFisher, Asse, Belgium) on a shaker for 1 h at 4 °C. After imidazole elution, the suspension was loaded on a size exclusion chromatography (SEC) column (HiLoad Superdex 200 pg, GE Healthcare, Machelen, Belgium), mounted on an AKTA avant high performance liquid chromatographer (HPLC) (GE Healthcare). Purity was evaluated on a 12% sodium dodecyl sulfate-polyacrylamide gel electrophoresis (SDS-PAGE) under reducing conditions, followed by staining with Coomassie blue. The presence of lipopolysaccharide (LPS) was measured using a limulus amebocyte lysate (LAL)-test (Endosafe^®^ nexgen-PTS™, Charles River, Ecully, France). Moreover, Nb R3B23, specific for 5T2 M-proteins [33], was produced as well and used during the study as negative control.

### 2.4. Evaluation of Affinity/Kinetics

The affinity for moLAG-3 of purified Nbs and the off-rate of Nbs in periplasmic extracts was performed using a Biacore T200 device (GE Healthcare, Machelen, Belgium). The measurements were performed at 25 °C using Hepes-buffered saline (HBS; 0.01 M HEPES pH 7.4, 0.15 M NaCl, 3 mM EDTA, 0.005% Tween 20) as running buffer. Briefly, recombinant, moLAG-3 protein was diluted to 10 μg/mL in 10 mM Na-acetate pH 5.5 and immobilized on a CM5 sensor chip using linkage chemistry with 1-(3-(dimethylamino)propyl)-3-ethylcarbodi-imide (EDC) and *N*-hydroxy-succinimide (NHS). Free EDC-NHS linkers present on the chip were neutralized with 1 M elthanolamine-HCl pH 8.5. Three dilutions of periplasmic extracts and seven dilutions of purified Nbs were analyzed at a flow rate of 10 µL/min. The association was set to 240 s and the dissociation to 300 s for each dilution. After each cycle, the chip was regenerated twice for 30 s using 100 mM Glycine HCl pH2.0. The measured response units from the flow channel on which moLAG-3 protein was immobilized was subtracted from the response units measured on a flow channel without protein. Additionally, the signal from the blank (HBS only) was subtracted from each measurement. As a result, the off-rate value k_off_ for Nbs in periplasmic extracts and the equilibrium dissociation constant K_D_ for purified Nbs were calculated using the Biacore T200 evaluation software (GE Healthcare, Machelen, Belgium).

### 2.5. Mice

Female, 6 to 12-week-old C57BL/6, B6.129S2-Lag3tm1Doi/J (moLAG-3 gene-deficient or knock-out (KO)) and Swiss Nude Crl:NU(Ico) Foxn1nu (immunodeficient) mice were purchased from Charles River (Ecully, France). All experiments using mice were approved by the Ethical Committee for laboratory animals of the Vrije Universiteit Brussel and executed in accordance to the European guidelines for animal experimentation (ethical dossier number 15-214-1).

### 2.6. Cell Lines

The TC-1 mouse lung epithelial cell line was kindly provided by T.C. Wu (Johns Hopkins University, Baltimore, MD, USA). The culture medium consisted of RPMI 1640 medium (Sigma-Aldrich, Zwijndrecht, Belgium), supplemented with 10% FCI serum (Harlan, Horst, The Netherlands), 2 mmol/L l-glutamine (L-Glu; Sigma-Aldrich, Zwijndrecht, Belgium), 100 U/mL penicillin, 100 µg/mL streptomycin (PS; Sigma-Aldrich, Zwijndrecht, Belgium), 1 mmol/L sodium pyruvate, and non-essential amino acids (Sigma-Aldrich, Zwijndrecht, Belgium), 12.5 mM d(+)-glucose, 1 mM Geneticin (G418), 5 mM hepes and 50 μM β-mercaptoethanol. Human embryonal kidney (HEK) 293T cells were obtained from the American Type Culture Collection (ATCC, Molsheim Cedex, France) and cultured in Dulbecco’s modified Eagle’s medium (DMEM) (Sigma-Aldrich, Zwijndrecht, Belgium) supplemented with 10% FBS (Harlan, Horst, The Netherlands), l-Glu, and PS.

### 2.7. Production of Lentiviral Vectors and Transduction of Cells

The transfer plasmid encoding moLAG-3 was generated using Gibson cloning. Briefly, gBlocks were designed to contain the coding sequence for moLAG-3, nucleotide 355 to 1920 from NM_008479.2, and purchased at Integrated DNA Technologies (IDT). The sequences were flanked by 20 nucleotides that allow creating overhangs with the donor lentiviral transfer vector pHR’, which together with the packaging plasmid pCMVΔR8.9 and the VSV.G encoding plasmid pMD.G, was a gift from D. Trono (University of Geneva, Switzerland). Accordingly, the production of lentiviral vectors and the transduction of HEK293T cells and TC-1 cells was performed as described [34]. HEK293T and TC-1 cells transduced with lentiviral vectors harboring moLAG-3 are referred to as 293T-LAG-3 cells and TC-1-LAG-3 cells respectively. Non-transduced cells are referred to as wild type (WT) cells.

### 2.8. Flow Cytometry

The procedure for staining of cell surface markers was previously described [35]. Flow cytometry experiments were performed on the LSRFortessa and FACSCelesta flow cytometer (BD Biosciences). Data were analyzed with FACSDiva (BD Biosciences) or FlowJo X^®^ (Tree star, Inc., Ashland, OR, USA) software.

### 2.9. Inoculation of Tumor Cells

Swiss nude Crl:NU(Ico) Foxn1nu mice were subcutaneously injected with 5 × 10^5^ TC-1-WT and TC-1-LAG-3 cells at their left and right flank respectively. The well-being of the mice was examined daily. Tumor growth was measured every other day using an electronic caliper and the tumor volume was calculated using the formula: (length × width^2^)/2 [28].

### 2.10. ^99m^Tc-Nb Labeling, Pinhole SPECT-Micro-CT Imaging and Image Analysis

The labeling of Nbs with ^99m^Tc was performed as previously described [36]. To summarize, ^99m^Tc-tricarbonyl [^99m^Tc(H_2_O)_3_(CO)_3_]^99m^ was complexed with the C-terminal HIS_6_-tag of the Nbs. The former was generated using the Isolink^®^ labelling kit (Mallinckrodt Medical BV, Petten, The Netherlands). The ^99m^Tc-Nb solution was subjected to two purification steps, i.e., gel filtration on a NAP-5 column (GE Healthcare, Machelen, Belgium) pre-equilibrated with PBS to remove uncomplexed (^99m^Tc(H_2_O)_3_(CO)_3_)^+^ and filtration through a 0.22 μm filter (Millipore, Haren, Belgium) to remove aggregates. Labeling efficiency was determined by instant thin-layer chromatography (iTLC) before and after purification, with 100% acetone as the mobile phase. Uncomplexed ^99m^Tc(H_2_O)_3_(CO)_3_ migrates with the mobile phase and separates from the complexed fraction (^99m^Tc-labeled Nb). The ratio between uncomplexed and complexed fraction determines the radiochemical purity. Imaging was performed as described previously [31]. Mice used for imaging experiments, were injected intravenously with 100 µL of ^99m^Tc-labeled Nbs (5 µg) with on average 51.1 ± 16.7 MBq of injected activity, 1 h before pinhole SPECT-micro-CT imaging. SPECT-CT imaging was performed using a Vector^+^ scanner from MiLABS. SPECT imaging was performed with a 1.5 mm 75-pinhole general purpose collimator, in spiral mode with six bed positions, the total body SPECT scan time was 15 min, 150 s per position. The total CT-scan time was 139 s, set to 60 kV and 615 mA. After imaging, mice were sacrificed and organs were isolated to evaluate the organ-specific uptake of the tracer using a γ-counter (Cobra Inspector 5003, Canberra, Packard, Illinois, IL, USA). The tissue/organ uptake was corrected for decay and calculated as the percentage of injected activity per gram tissue (%IA/g). Image analysis was performed using HOROS medical imaging viewer (LGPL license at Horosproject.org) and AMIDE (Medical Image Data Examiner software, UCLA, California, CA, USA).

### 2.11. Single Cell Preparation of Organs and Tumors

Single cell suspensions of lymph nodes, spleens, and implanted tumors were prepared according to the protocols of Miltenyi Biotec. Briefly, tumors were cut in pieces of approximately 5 mm and transferred to gentleMACS C tubes containing 5 mL ice-cold RPMI 1640 supplemented with 1000 U/mL DNAse I. The tumors were homogenized on the gentleMACS dissociator. The suspension was filtered through a 70 μm nylon filter (BD Falcon) and centrifuged for 5 min at 1500 rpm. Erythrocytes were lysed by resuspending the cell pellet with 5 mL red blood cell lysis buffer (0.16 M NH4Cl, 0.17 M Tris, pH 7.2). After 3 min incubation, the buffer was neutralized by adding 10 mL phosphate buffered saline (PBS, Sigma-Aldrich). Cells were centrifuged and counted (CasyTon, Innovatis, Reutlingen, Germany) and prepared for flow cytometry staining and analysis. Lymph nodes and spleens were prepared by mincing the organ through a 70 μm nylon filter in 5 mL PBS containing 1000 U/mL DNAse I (StemCell). Cells were centrifuged and incubated with 5 mL red blood cell lysis buffer. After 3 min of incubation, the buffer was neutralized by adding 10 mL PBS. Cells were centrifuged and counted (CasyTon) and prepared for flow cytometry staining and analysis.

### 2.12. Immunohistochemistry

Organs were incubated in 4% formalin for 24 h before transfer to the tissue processing apparatus Histokinette (Leica TP 1028) where the tissues are paraffin embedded. Slices of 3 μm were prepared with the microtome and baked at 60 °C before staining. Hematoxylin eosin saffron (HES) staining was performed following standard protocols. Stained sections were mounted with mounting media (Pertex, for histolab) and scanned with an Aperio digital pathology slide scanner (Leica Aperio CS2). Immunofluorescence (IF) staining of moLAG-3 was performed using an anti-LAG-3 antibody (Abcam, EPR20294-77). Therefore, the sections were deparaffinized, rehydrated and antigen retrieval was performed in citrate buffer pH 6.0 using the 2100 Retriever (Aptum Biologics). Slices were washed with PBS, blocked with serum free Dako Protein block (Agilent Technologies), and incubated overnight with the primary anti-LAG-3 antibody at 4 °C in a humidity chamber. After three wash steps with PBS, the secondary antibody (anti-rabbit AF477/Cy5, Jackson Laboratories) was added for 1 h. After three washes in PBS, the sections were mounted with fluorescent mounting medium containing 4′,6-diamidino-2-phenylindole (DAPI) (Agilent Technologies). For spleen sections, an autofluorescence quenching kit was used to lower the autofluorescence of red blood cells (Vector TrueVIEW, SP-8400). The stained sections were evaluated with the EVOS FL Auto fluorescence microscope (Life Technologies, Asse, Belgium).

### 2.13. Statistics

All statistical analyses were performed with GraphPad Prism software. All data are represented as mean ± standard deviation (SD). *p*-values were calculated using a Kruskal–Wallis test followed by a Dunn’s post hoc test or using a two-way Anova followed by a Tukey post hoc test. The asterisks in the figures indicate statistical significance as follows: * *p* < 0.05; ** *p* < 0.01; *** *p* < 0.001; **** *p* < 0.0001; n.s. not significant.

## 3. Results

### 3.1. Generation and Identification of Nbs Specific for Mouse LAG-3

**Figure 1:**
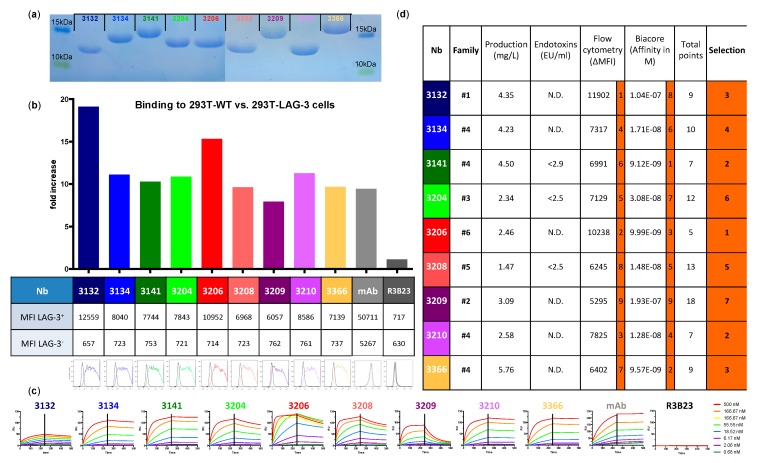
Characterization of binding and affinity for mouse LAG-3 (moLAG-3) of purified nanobodies (Nbs). (**a**) sodium dodecyl sulfate-polyacrylamide gel electrophoresis (SDS-PAGE) of 20 μg purified Nb stained with Coomassie blue (*n* = 1). (**b**) Binding of 1 μg of His_6_-tagged Nbs on 293T-wild type (WT) cells versus 293T-moLAG-3 cells (*n* = 1). The graph shows the ratio of bound nanobody on 293T-WT versus 293T-LAG-3, while the table shows the mean fluorescence intensities used to calculate the ratio and the flow cytometry histograms show the binding of the nanobody to 293T-WT (black line) and to 293T-LAG-3 (matched colored line) (*n* = 1). (**c**) Evaluation of affinity/kinetics of purified Nbs on immobilized recombinant moLAG-3 protein (*n* = 2). Each sensogram represents the binding of a 3-fold dilution series (0.68, 2.06, 6.17, 18.5, 55.6, 167, and 500 nM) of the same Nb. (**d**) Summary of Nb characterization. Selection is based on binding capacity to moLAG-3 evaluated by flow cytometry and surface plasmon resonance (SPR)-measured affinity. The columns in orange show a ranking per evaluated criterium and an overall ranking for each Nb clone.

The generation of Nbs, targeting moLAG-3, was initiated by immunizing llamas with recombinant mouse and human LAG-3 proteins. From llama lymphocytes, VHH encoding sequences were PCR-amplified and cloned in a phagemid vector to create immune VHH-libraries in *E. coli*. The libraries were phage-displayed and subjected to several rounds of panning on recombinant mouse or human LAG-3 proteins that were immobilized on immunosorbent plastic. Periplasmic extracts from random clones were analyzed using ELISA for binding to LAG-3 recombinant protein. The selected libraries resulted in 37 different Nbs for human LAG-3 and 26 different Nbs for mouse LAG-3 belonging to 10 and nine different complementarity-determining region (CDR) groups respectively. The 26 Nbs recognizing moLAG-3 did not cross-react with human LAG-3. Periplasmic extracts of moLAG-3 Nbs were additionally tested in flow cytometry on lentivirally transduced HEK293T cells that overexpress moLAG-3 and in off-rate screenings using SPR on immobilized recombinant moLAG-3 protein (Supplementary Figure S1a–c). As such, nine moLAG-3-binding Nbs from six different sequence families were selected, produced, and purified as HIS_6_-tagged proteins from cultured *E. coli* cells. R3B23 Nb, specific for the M-protein of the 5T2 multiple myeloma mouse model, was used as negative control throughout the study [33]. The purity was assessed using SDS-PAGE (Figure 1a) and LPS content was measured using a LAL-test (Figure 1d). Finally, the binding of the purified Nbs was evaluated by flow cytometry on moLAG-3 expressing cells (Figure 1b,d) and affinity was further assessed using SPR, ranging from 9.12 to 193 nM (Figure 1c,d). The characterization and ranking of the nine purified Nbs are summarized in Figure 1d.

### 3.2. Imaging of Mouse LAG-3 in Naive C57BL/6 Mice

**Figure 2:**
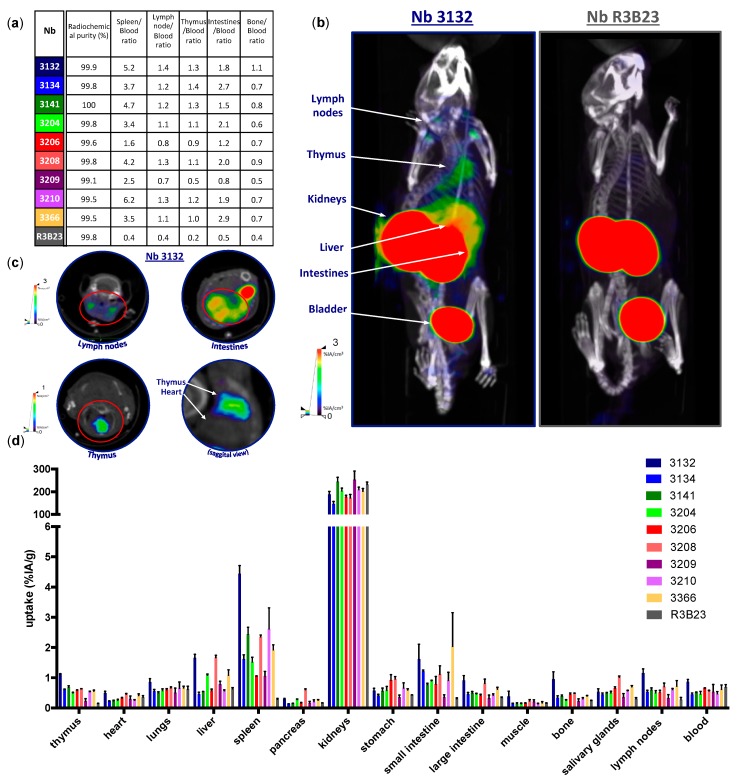
Single photon emission computed tomography (SPECT)/CT imaging and biodistribution of Nbs in naive C57BL/6 mice. (**a**) Radiochemical purity of in vivo injected ^99m^Tc-Nb conjugates and the ratio between the uptake of the Nbs in the spleen, thymus, intestines, lymph nodes, and bones with the uptake values found in the blood (*n* = 1). (**b**,**c**) SPECT/CT imaging of mice 60 min after intravenous injection of ^99m^Tc-labeled anti-moLAG-3 Nbs 3132 and 3206, showing specific signals in the lymph nodes, intestines, thymus, and bone marrow as compared to control Nb R3B23. The data shown are representative for three mice (*n* = 1). (**d**) Ex vivo γ-counting of the isolated organs from healthy C57BL/6 mice 80 min after injection with ^99m^Tc-labeled anti-moLAG-3 Nbs or control Nb R3B23. The graph summarizes the biodistribution of the Nbs in three mice, expressed as mean ± SD of percentage of injected activity per gram of organ or tissue (%IA/g) (*n* = 1).

**Figure 3:**
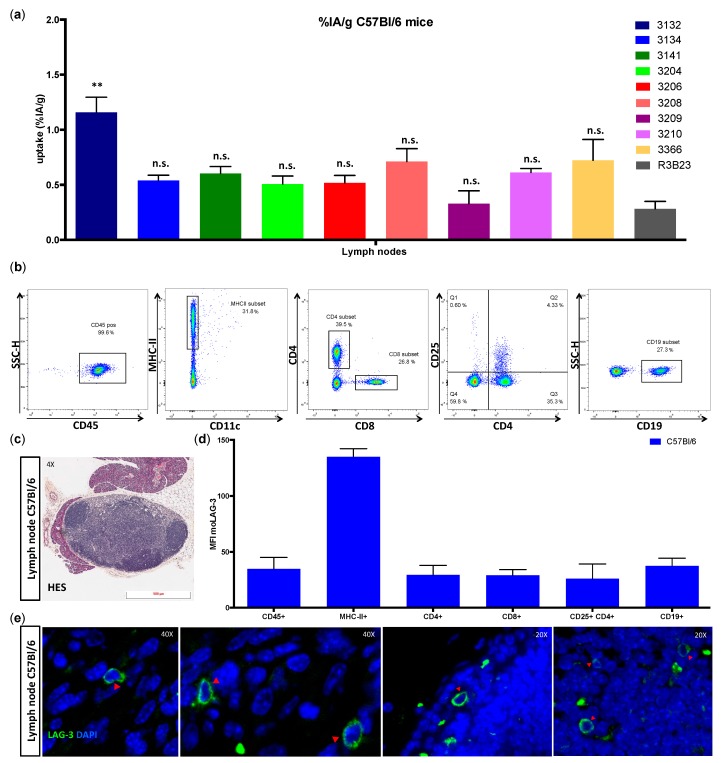
Ex vivo analysis of immune cell populations within submandibular lymph nodes of C57BL/6 mice. (**a**) The graph summarizes the uptake of radiolabeled Nbs in submandibular lymph nodes 80 min after injection of three naive C57BL/6 mice as mean ± SD (*n* = 1). (**b**) Flow cytometry gating for single cell suspensions prepared from isolated submandibular lymph nodes. (**c**) Hematoxylin eosin saffron (HES) staining of formalin-fixed, paraffin-embedded (FFPE) submandibular lymph node section. The data shown are representative for three independent mice (*n* = 1). (**d**) Expression of moLAG-3 (mean fluorescence intensity, MFI) on different immune cell subsets analyzed by flow cytometry. The graph summarizes the results of three mice as mean ± SD (*n* = 1). (**e**) Immunofluorescence microscopy on a lymph node section, stained with moLAG-3 (green) and 4’,6-diamidino-2-phenylindole (DAPI, blue). The red arrows show moLAG-3 expressing cells. The data shown are representative for three independent mice (*n* = 1).

**Figure 4:**
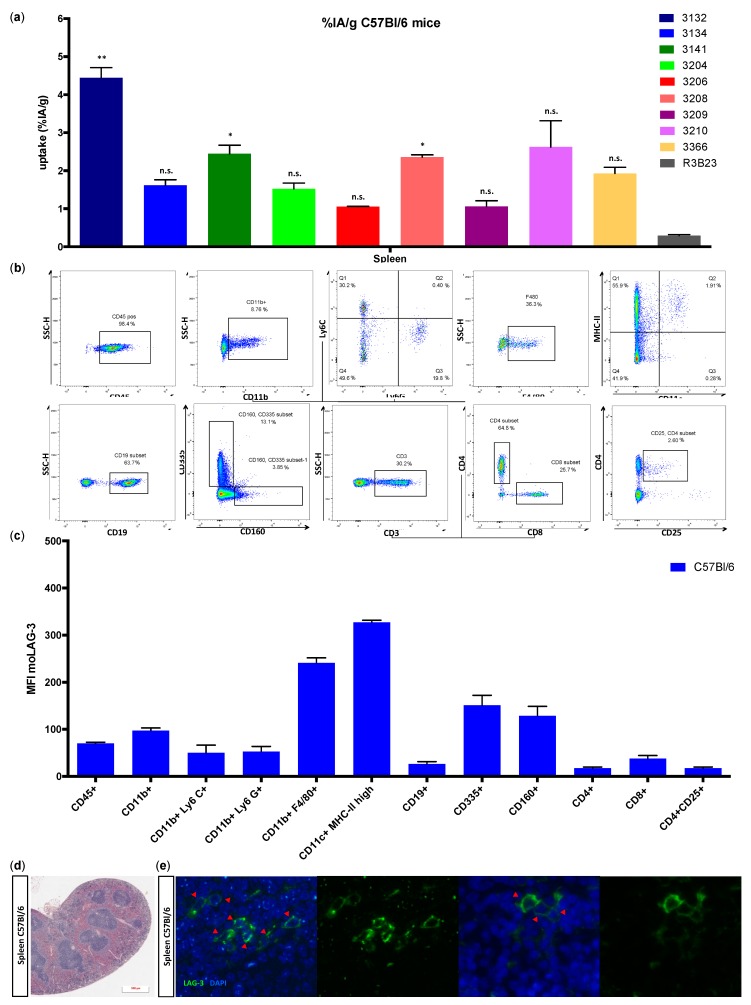
Ex vivo analysis of immune cell populations within the spleen of C57BL/6 mice. (**a**) Uptake of the radiolabeled Nbs in the spleen 80 min after injection of naive C57BL/6 mice. The graph summarizes the results of three mice as mean ± SD (*n* = 1). (**b**) Flow cytometry gating for single cell suspensions prepared from isolated spleens. The data shown are representative for three independent mice (*n* = 1). (**c**) HES staining of FFPE spleen sections. The data shown are representative for three independent mice (*n* = 1). (**d**) Expression of moLAG-3 (MFI) on different immune cell subsets analyzed by flow cytometry. The graph summarizes the results of three mice as mean ± SD (*n* = 1). (**e**) Immunofluorescence microscopy on a spleen section, stained with moLAG-3 (green) and DAPI (blue). The red arrows show moLAG-3 expressing cells. The data shown are representative for three independent mice (*n* = 1).

Selected moLAG-3-specific Nbs were radiolabeled with ^99m^Tc at their HIS_6_-tail and radioconjugates were purified by SEC and filtration. Radiochemical purity was assessed using iTLC and was consistently >99% (Figure 2a). Naive C57BL/6 mice were injected intravenously with 100 μL/5 μg of ^99m^Tc-labeled Nbs with on average 60.7 ± 8.1 MBq coupled activity. At 60 min post injection, mice were anesthetized, and SPECT and CT images were acquired (Figure 2b,c). Subsequently, the mice were sacrificed, and organs were isolated for γ-counting in order to evaluate the uptake and biodistribution of radiolabeled Nbs in specific anatomic regions (Figure 2d). As a result of renal clearance, Nbs mostly accumulate in the kidneys and the bladder. The amount of Nbs remaining in the blood is low and to a comparable amount as the blood accumulation of the control Nb R3B23. Compared to the uptake of R3B23, anti-moLAG-3 Nbs 3132, showed significant higher uptake in the lymph nodes (*p* = 0.0389) (Figure 3). Moreover, compared to the uptake of R3B23, anti-moLAG-3 Nbs 3132, 3141 and 3208 showed significant higher uptake in the spleen (*p* = 0.0017) (Figure 4) and thymus (*p* = 0.0020) (data not shown). Similarly, Nbs 3132 and 3134 showed significant higher uptake in the small intestines (*p* = 0.0255) (data not shown). Additionally, Nb 3132 showed significant higher uptake in the bone (*p* = 0.0064) (data not shown) The ratio between the uptake of the Nbs in the spleen, thymus, lymph nodes, small intestines, and bone with the uptake values in the blood were calculated (Figure 2a). Due to the high signals in the kidneys, the uptake observed in the spleen could not be visualized on SPECT/CT images since these organs are in close proximity to each other. In contrast, the signal from the intestine, lymph nodes, and thymus were visible. This suggested the presence of moLAG-3 expressing immune cells in these organs. LAG-3 Nbs 3132, 3204, 3208, and 3366 but not others showed elevated uptake in the liver.

### 3.3. Ex Vivo Evaluation of LAG-3 Expression on Immune Cells

In order to examine the source of the radioactivity levels found in the lymph nodes of naive C57BL/6 mice (Figure 3a), we harvested the submandibular lymph nodes and evaluated moLAG-3 expression on different immune cell populations from single cell preparations by flow cytometry. The gating strategy is illustrated in Figure 3b. Although very low in mean fluorescence intensities, the expression of moLAG-3 could be detected on lymphocytes like CD4^+^ T cells, CD8^+^ T cells, and CD19^+^ B cells. In contrast, higher expression levels of moLAG-3 could be detected on MHC-II^+^ antigen presenting cells (Figure 3d). In order to show the intact structure of the lymph node section, we performed a HES staining (Figure 3c). Subsequently, we performed immunofluorescence IHC to detect moLAG-3 expressing cells on lymph node sections (Figure 3e).

The ex vivo measured uptake of ^99m^Tc-moLAG-3 Nbs showed a significantly higher uptake of Nb 3132, 3141, and 3208 in the spleen compared to the control Nb R3B23 (Figure 4a). The specificity of this signal was corroborated by the detection of high levels of moLAG-3 expression in single cell preparations of the spleen (Figure 4c,d). The gating strategy is illustrated in Figure 4b. In order to show the intact structure of the spleen section, we performed a HES staining (Figure 4d). Subsequently, we performed immunofluorescence microscopy on the spleen sections to visualize moLAG-3 expressing cells (Figure 4d).

### 3.4. Imaging in Naive LAG-3 Gene-Deficient Mice

**Figure 5:**
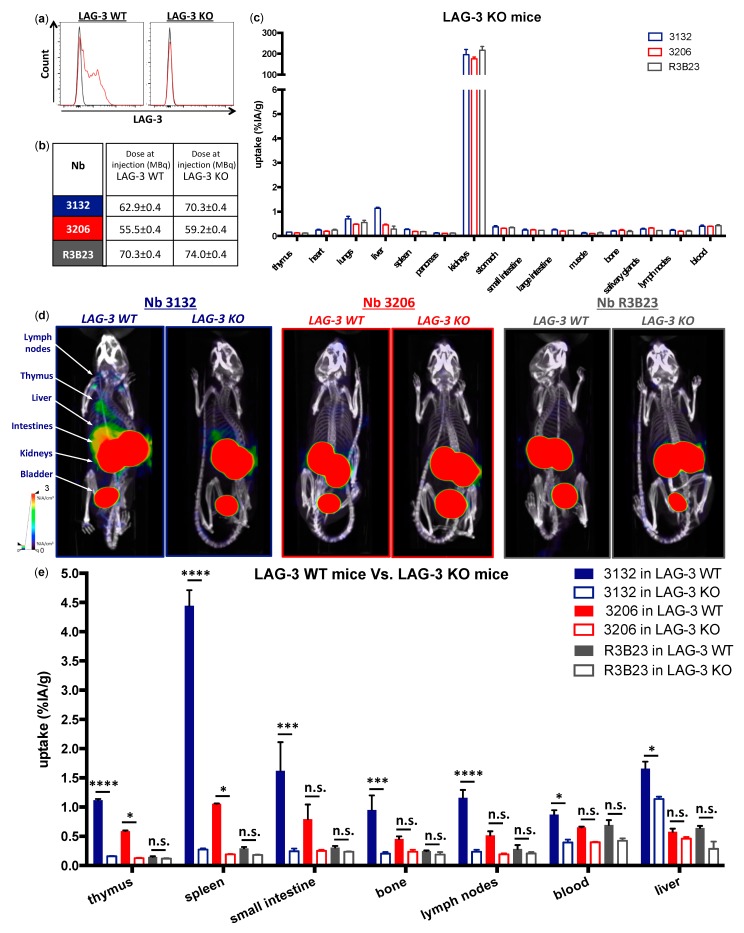
Characterization and in vivo SPECT/CT imaging with anti-moLAG-3 Nbs in LAG-3 knock-out (KO) mice. (**a**) Flow cytometry analysis of moLAG-3 expression on stimulated lymphocytes from LAG-3 WT and LAG-3 KO mice. The flow cytometry graphs are representative for three independent mice (*n* = 1). (**b**) Dose of in vivo injected ^99m^Tc-Nb conjugates in LAG-3 WT or LAG-3 KO mice. (**c**) Ex vivo γ-counting of isolated organs from LAG-3 KO mice 80 min after injection of ^99m^Tc-labeled Nbs targeting moLAG-3 or control Nb R3B23. The graph summarizes the data obtained in three mice as mean ± SD of %IA/g (*n* = 1). (**d**) Representative SPECT/CT scans of LAG-3 WT mice (left) and LAG-3 KO mice (right) 60 min after injection of ^99m^Tc-labeled Nb 3132, 3206, or control Nb R3B23. (**e**) Ex vivo γ-counting of isolated organs from LAG-3 WT mice and LAG-3 KO mice 1 h after injection of ^99m^Tc-labeled Nbs targeting moLAG-3 or control Nb R3B23. The graph summarizes the data obtained in three mice as mean ± SD of %IA/g (*n* = 1).

We next evaluated the biodistribution profile of ^99m^Tc-moLAG-3 Nbs 3132 and 3206 in LAG-3 KO mice, to confirm the specificity of the uptake observed in naive C57BL/6 mice. In order to confirm that LAG-3 expression is absent on immune cells from LAG-3 KO mice, we isolated spleens from LAG-3 KO mice and prepared single cell suspensions as described above. The spleen cells were stimulated in vitro with CD3/CD28 dynabeads for 24 h in order to activate them [8,37]. For comparison, spleen cells from naive C57BL/6 mice, referred to as LAG-3 WT, were treated in the same way. LAG-3 expression was observed on lymphocytes from LAG-3 WT mice and not on lymphocytes from LAG-3 KO mice (Figure 5a). This suggests that our LAG-3 targeting Nbs should not be able to detect any LAG-3 in vivo. To evaluate this, we intravenously injected LAG-3 KO mice with ^99m^Tc-labeled Nbs (Figure 5b) and performed SPECT/CT imaging (Figure 5d) and biodistribution analysis (Figure 5c) under identical conditions as in LAG-3 WT mice. We compared two moLAG-3 specific Nbs for this experiment: Nb 3132 had a significantly higher uptake in the spleen, thymus, lymph nodes and small intestines, from naive C57BL/6 mice, compared to the control Nb R3B23, although in vitro its measured affinity was only modest. Contrarily, the measured affinity of Nb 3206 for moLAG-3 was much higher while the elevated uptake in lymphoid organs versus R3B23 was only modest. Comparative uptake levels in LAG-3 WT versus KO mice are depicted in Figure 5e. The uptake of ^99m^Tc-labeled Nb 3132 in the liver was significantly lower when injected in LAG-3 KO mice compared to LAG-3 WT mice (*p* < 0.05, data not shown), suggesting that this accumulation was specific to a certain extent. Moreover, compared to the uptake of Nb 3132 in LAG-3 WT mice, the uptake in LAG-3 KO mice was significantly decreased in organs like the thymus (*p* < 0.0001), spleen (*p* < 0.0001), small intestines (*p* < 0.001), bone (*p* < 0.001), lymph node (*p* < 0.0001), and blood (*p* < 0.05). LAG-3-specific organ uptake values for Nb 3206 only reached statistical significance in the thymus and spleen.

### 3.5. Specific Targeting of Mouse LAG-3 Expressed in the Tumor by Mouse LAG-3 Specific Nbs

**Figure 6:**
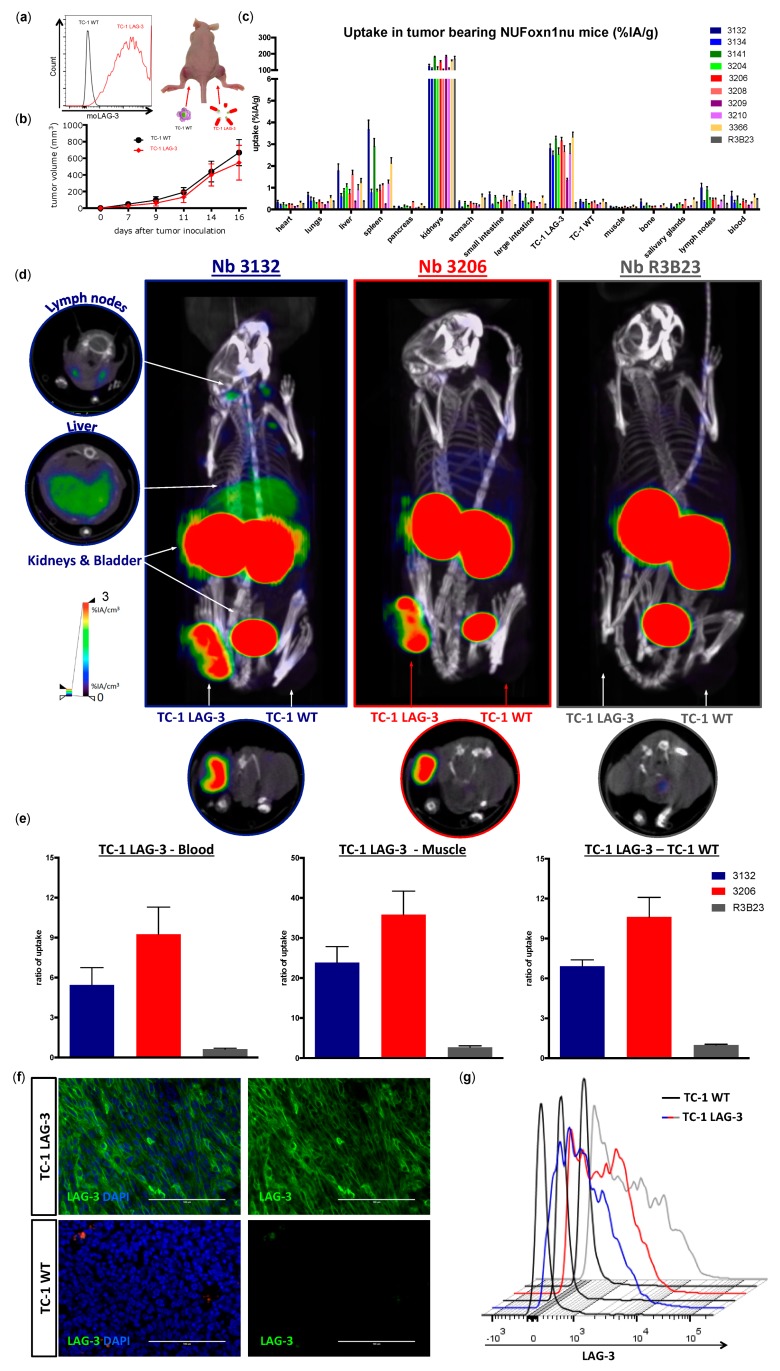
SPECT/CT imaging and biodistribution analysis of ^99m^Tc-labeled anti-moLAG-3 Nbs in nude mice bearing either TC-1-LAG-3 or TC-1-WT tumors. (**a**) Flow cytometry analysis of the moLAG-3 expression of lentivirally-modified TC-1 cells (red line) or unmodified TC-1 cells (black line). (**b**) Graph showing the tumor growth of TC-1-WT cells (black line) or TC-1-LAG-3 cells (red line). The graph summarizes the data as mean ± SD of 30 mice per condition (*n* = 1). (**c**) Ex vivo γ-counting of isolated organs from tumor bearing nude mice 80 min after injection of ^99m^Tc-labeled Nbs targeting moLAG-3 or control Nb R3B23. The graph summarizes the data obtained in three mice as mean ± SD of uptake, expressed as %IA/g (*n* = 1). (**d**) Representative SPECT/CT scans of three TC-1-LAG-3 (right flank) and TC-1-WT (left flank) tumor bearing mice 60 min after injection of ^99m^Tc-labeled Nb 3132, 3206, or control Nb R3B23 (*n* = 1) (**e**) As a measure of image contrast, TC-1-LAG-3 tumor-to-blood, TC-1-LAG-3 tumor-to-muscle, and TC-LAG-3 tumor to TC-1-WT tumor ratios are determined. The graph summarizes the data obtained in three mice as mean ± SD (*n* = 1). (**f**) Representative immunofluorescence microscopy of sections from dissected TC-1-LAG-3 tumors and TC-1-WT tumors derived from three mice (*n* = 1). moLAG-3 was detected using an anti-moLAG-3 mAb (green). The nuclei of the cells were stained with DAPI (blue). (**g**) Flow cytometry analysis of moLAG-3 expression on single cell preparations of TC-1-LAG-3 (blue, red, and grey lines for Nbs 3132, 3106, and R3B23 respectively) versus TC-1-WT tumors (black lines). The flow histogram graph is representative for three mice (*n* = 1).

We next wondered whether ^99m^Tc-labeled Nbs target moLAG-3 in tumors and can discriminate between moLAG-3 expressing tumors and those that lack moLAG-3 expression. Therefore, we lentivirally transduced TC-1 lung epithelial cells to express moLAG-3, referred to as TC-1 LAG-3. The non-transduced TC-1 cells served as a control and are referred to as TC-1 WT (Figure 6a). We subcutaneously implanted the TC-1 cells in immunocompromised mice. As shown in Figure 6a, the right flank of the mice was injected with TC-1 LAG-3 cells and the left flank with TC-1 WT cells. The tumor growth was evaluated every other day and no difference was observed between the growth of TC-1 WT and TC-1 LAG-3 tumors (Figure 6b). The nine selected Nbs were radiolabeled with ^99m^Tc and mice were intravenously injected with ^99m^Tc-labeled Nbs with an average activity of 41.1 ± 15.3 MBq (Figure 6c). SPECT/CT images were acquired 1 h post injection followed 20 min later by the dissection of organs, tissues, and tumors and the measurement of radioactivity levels (Figure 6c,d). Ex vivo analysis allowed us to further classify the Nbs suited for imaging, based on the different signal ratios. We compared specific uptake of the different Nbs in the TC-1 LAG-3 with TC-1 WT tumors, blood, muscle, and liver, and calculated the ratios in order to compare the uptake to background activity (Figure 6e). All anti-moLAG-3 Nbs were convincingly able to discriminate LAG-3 expressing from LAG-3 non-expressing tumors by nuclear imaging. Subsequently, representative images of Nbs 3132 and 3206 are shown in Figure 6d. Moreover, dissected TC-1 WT and TC-1 LAG-3 tumors were analyzed for moLAG-3 expression by flow cytometry and IHC and showed the persistent moLAG-3 expression after in vivo growth (Figure 6f,g).

## 4. Discussion

Immune checkpoint therapy has become an important pillar in cancer therapy with mAbs targeting CTLA-4 and PD-1/PD-L1 showing clinical benefit in patient subgroups with solid tumors. Despite its unseen success, a significant number of patients do not respond to anti-CTLA-4 and/or PD-1/PD-L1 therapy, and inhibition of more recently described inhibitory receptors such as LAG-3 is actively investigated in the clinic as an alternative or additional treatment [10,14,15]. As the list of immune checkpoint inhibitors and their combination regimens grow, there is an increasing need for easy-to-implement strategies that can predict patient response and that allow monitoring of patient responses during therapy. Given the current prizes of immune checkpoint drugs, the development of predictive markers is not only clinically relevant but also an economic necessity.

Detection of expression of immune checkpoints has mainly been performed by IHC. However, IHC provides a static picture at time of biopsy and is therefore a less suitable technique to predict therapy outcome even if the tumor is accessible. Limitations of IHC for predictive testing have been extensively discussed by Broos et al. [21]. In contrast to IHC, molecular imaging is a noninvasive technique that can be performed repeatedly and allows quantification of tumor characteristics irrespective of the location of the tumor lesions. It was recently shown that PET is able to detect expression of the immune checkpoint receptor PD-1 and its ligand PD-L1, and that PET images correlated better with therapy outcome than IHC data [22,23].

Different moieties can be used for noninvasive molecular imaging. However, it is generally accepted that small antigen binding moieties will generate fast and high-contrast images, as radiolabeled nanobodies were shown to be cleared from the blood as fast as 20–30 min after intravenous injection [38,39]. As LAG-3 blocking mAbs are currently tested in 28 different clinical trials for the treatment of cancer, we evaluated Nbs specific for the inhibitory immune checkpoint LAG-3 and used these for noninvasive imaging of moLAG-3.

We showed that lymphocytes, residing in the lymph nodes of naive and healthy C57BL/6 mice, express low levels of moLAG-3. In contrast, lymph node residing antigen presenting cells expressed higher levels of moLAG-3. Within the spleen, the immune cell population (CD45^+^) with the highest moLAG-3 expression could be detected on antigen-presenting cells, like dendritic cells and macrophages, and on NK and NKT cells [4]. Moreover, very low amounts of moLAG-3 are expressed on CD19^+^ B cells, and CD4^+^ and CD8^+^ T cells, which is comparable to the low levels found on lymphocytes isolated from lymph nodes. Nevertheless, out of nine different ^99m^Tc-labeled anti-moLAG-3 Nbs, Nb 3132 was still able to detect and visualize low levels of moLAG-3 expressing immune cells residing in the spleen and lymph nodes. Although the affinity of Nb 3132 for moLAG-3 is rather low, injection of ^99m^Tc-labeled Nb 3206, with a 10-fold higher affinity for moLAG-3, was less able to detect and image moLAG-3 expressing cells in the spleen and lymph nodes. Interestingly, when evaluating our Nbs for binding on moLAG-3 expressing cells using flow cytometry, Nb 3132 showed the highest binding of all Nbs. This observation could mean that recombinant moLAG-3 coated chips for SPR or membrane expressed LAG-3 for flow cytometry analysis are not completely identical and thus could lead to under or overestimation of Nb affinity. Moreover, the affinity of Nbs is only one of the parameters used to predict its potential for imaging purposes. Vaneycken et al. showed that the dissociation rate of Nbs did not correlate with tumor uptake since their Nb with the slowest dissociation rate targeted poorly to tumors, while another Nb, with a fast dissociation rate, targeted very well [40]. Nevertheless, the specific uptake for ^99m^Tc-labeled anti-moLAG-3 Nb 3132 and 3206 was further validated using LAG-3 KO mice, where no uptake could be detected in organs like spleen and lymph nodes, expect for the liver. However, the uptake of ^99m^Tc-labeled Nb 3132 in the liver of LAG-3 KO was significantly lower compared than in LAG-3 WT mice, suggesting that accumulation was partly due to specific binding. To this date, no supportive data could be found in literature describing LAG-3 expression in the liver. Several studies have reported non-specific uptake of radiolabeled Nbs in the liver, explaining the remaining liver uptake in LAG-3 KO mice [39]. Unfortunately, the nonspecific accumulation in the liver could obscure possible liver metastases and such accumulation should be taken into account when selecting Nbs for the noninvasive imaging of human LAG-3 since these are most likely to reach the clinic compared to moLAG-3 targeting Nbs.

Next, we evaluated the potential of the ^99m^Tc-labeled Nbs to detect moLAG-3 present in dense tissues like tumors. For this purpose, we used an artificial model where mice, harboring a subcutaneous tumor that was lentivirally modified to overexpress moLAG-3, are imaged using our ^99m^Tc-labeled Nbs. Moreover, athymic nude mice were used for these experiments in order to prevent rejection of the lentiviral transformed TC-1 tumors. The uptake of Nb 3132 and 3206 in the moLAG-3 expressing tumors was comparable and with high contrast levels as fast as 1 h after injection. This shows that Nbs are excellent candidate molecules for the visualization of immune checkpoints within the tumor environment, like previously described in Broos et al. using PD-L1 targeting Nbs [31]. Moreover, in view of repeated imaging, which could be interesting for follow up of the immune checkpoint status during therapy, it is important to note that Nbs are weakly immunogenic because of their high similarity with human variable heavy chain fragments properties (reviewed by [24]).

These results allow us to further investigate the potential of noninvasive LAG-3 detection within the tumor environment using the selected Nb 3132. Furthermore, we will also continue with the development of human LAG-3 specific Nbs for the translation of this approach to the clinic. Taken together we are confident that small imaging agents, such as the here presented LAG-3 targeting Nbs, could be used as a diagnostic tool to noninvasively evaluate LAG-3 expression before and after immunotherapy and to adapt treatments accordingly.

## 5. Conclusions

With this report, we are the first to describe the use of Nbs targeting the immune checkpoint LAG-3 in the field of immuno-oncology. A panel of anti-LAG-3 Nbs was investigated using various techniques in order to identify the most optimal targeting moiety for the molecular imaging of LAG-3 expression in the tumor microenvironment. Nb 3132 was selected as the lead compound and has provided proof-of-concept that molecular whole-body imaging of LAG-3 expression is possible. These findings warrant further research into the noninvasive detection of LAG-3 on tumor infiltrating immune cells, since its expression has been related to poor prognosis in different cancer types [9,10]. The development of a Nb targeting human LAG-3 could enable LAG-3 imaging in cancer patients, which could be used for patient stratification and predicting therapeutic outcome of LAG-3 targeting cancer treatments [8,12,15].

## Supplementary Materials

The following are available online at https://www.mdpi.com/2218-273X/9/10/548/s1, Figure S1. Initial screening of 26 Nbs (periplasmic extracts) targeting moLAG-3. (**a**) Table summarizing the different selection steps i.e., ELISA, flow cytometry, and SPR. (**b**) Binding of 50 μL periplasmic extract on 293T-WT (grey line) versus 293T-LAG-3 cells (red line). (**c**) Evaluation of kinetics of periplasmic extract of the Nbs on immobilized Fc-tagged recombinant LAG-3 protein. Each sensogram represents a different concentration of the same Nb i.e., undiluted extract (red line), 1/4 diluted extract (orange line), 1/8 diluted extract (blue line), and a blank run (grey line). Nanobodies depicted in orange were further evaluated.
