# Supplementary material for: Noninvasive Imaging of the Immune Checkpoint LAG-3 Using Nanobodies, from Development to Pre-Clinical Use
Source: Biomolecules. 2019 Sep 29;9(10):548. doi: 10.3390/biom9100548 (PMC6843898; doi:10.3390/biom9100548)
Supplement: Supplementary file 1 [file biomolecules-09-00548-s001.pdf]

(**a**)

Nb	Family	ELISA		Flow cytometry (MFI)		Biacore (Off-rate)
		moLAG-3	Blank	293T-LAG-3	293T-WT	
3131	#4	+	−	2323	303	2.38E-03
3132	#1	+++	−	2089	309	2.29E-05
3133	#9	+++	−	1010	304	8.28E-06
3134	#4	+++	−	4380	304	1.70E-03
3135	#4	+	−	4663	312	1.57E-03
3136	#4	++	−	4024	315	1.73E-03
3137	#4	++	−	2469	307	1.63E-03
3138	#4	+++	−	4504	312	4.08E-03
3139	#4	++	−	2231	314	1.79E-03
3140	#4	++	−	1200	323	5.16E-03
3141	#4	+++	−	3226	319	2.47E-03
3142	#4	++	−	2762	312	7.14E-04
3143	#4	+++	−	1701	317	2.63E-03
3144	#4	++	−	2430	328	1.66E-03
3145	#4	+	−	1737	311	1.42E-03
3204	#3	+	−	1306	361	1.77E-03
3205	#4	+	−	2362	394	3.21E-03
3206	#6	+++	−	1363	450	1.52E-03
3207	#7	+	−	963	463	/
3208	#5	++	−	5645	374	/
3209	#2	+	−	2583	375	1.07E-02
3210	#4	+++	−	1699	408	1.81E-03
3212	#8	+++	−	1190	370	1.96E-03
3213	#4	+++	−	3386	392	2.83E-03
3366	#4	+	−	3914	608	9.57E-04
3371	#4	+	−	2145	574	7.51E-03

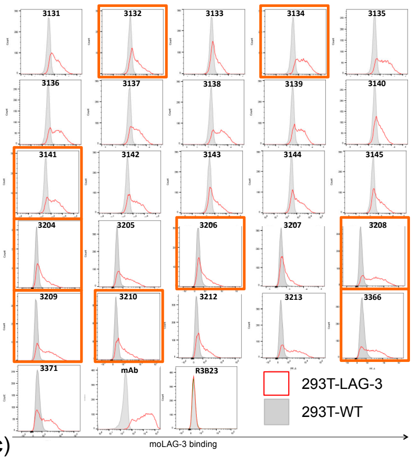

(**c**)

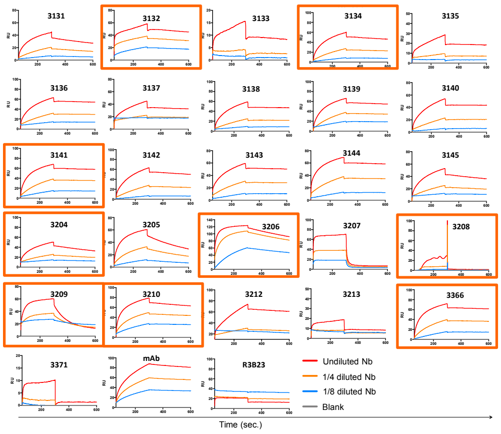

Supplementary Figure 1. Initial screening of 26 Nbs (periplasmic extracts) targeting moLAG-3. (**a**) Table summarizing the different selection steps i.e., ELISA, flow cytometry, and SPR. (**b**) Binding of 50 μL periplasmic extract on 293T-WT (grey line) versus 293T-LAG-3 cells (red line). (**c**) Evaluation of kinetics of periplasmic extract of the Nbs on immobilized Fc-tagged recombinant LAG-3 protein. Each sensogram represents a different concentration of the same Nb i.e., undiluted extract (red line), 1/4 diluted extract (orange line), 1/8 diluted extract (blue line), and a blank run (grey line). Nanobodies depicted in orange were further evaluated.
